# Comparison of minimally invasive transforaminal lumbar interbody fusion and midline lumbar interbody fusion in patients with spondylolisthesis

**DOI:** 10.1186/s13018-024-04764-2

**Published:** 2024-05-09

**Authors:** Yang-Yi Wang, Yu-Hsuan Chung, Chun-Hsien Huang, Ming-Hsien Hu

**Affiliations:** 1 Department of Orthopaedic Surgery, Show Chwan Memorial Hospital, No. 542, Sec 1, Chung-Shan Rd., Changhua, 500 Taiwan; 2 PhD Program in Translational Medicine, National Chung Hsing University, Taichung, Taiwan; 3 Rong Hsing Research Center for Translational Medicine, National Chung Hsing University, Taichung, Taiwan; 4 Department of Orthopaedic Surgery, Chang Bing Show Chwan Memorial Hospital, No.6, Lugong Rd., Lukang Township, Changhua County 505, Changhua, Taiwan; 5 Department of Post-Baccalaureate Medicine, College of Medicine, National Chung Hsing University, Taichung, Taiwan

**Keywords:** Cortical bone trajectory, Midline lumbar fusion, Minimally invasive procedure, Spondylolisthesis, Transforaminal lumbar interbody fusion

## Abstract

**Background:**

This study aimed to compare surgical outcomes, clinical outcomes, and complications between minimally invasive transforaminal lumbar interbody fusion (MIS TLIF) and midline lumbar interbody fusion (MIDLIF) in patients with spondylolisthesis.

**Methods:**

This study retrospectively compared the patients who underwent MIS TLIF (*n* = 37) or MIDLIF (*n* = 50) for spinal spondylolisthesis. Data of surgical outcomes (postoperative one-year fusion rate and time to bony fusion), clinical outcomes (visual analog scale [VAS] for pain and Oswestry Disability Index [ODI] for spine function), and complications were collected and analyzed.

**Results:**

There was more 2-level fusion in MIDLIF (46% vs. 24.3%, *p* = 0.038). The MIS TLIF and MIDLIF groups had similar one-year fusion rate and time to fusion. The MIDLIF group had significantly lower VAS at postoperative 3-months (2.2 vs. 3.1, *p* = 0.002) and postoperative 1-year (1.1 vs. 2.1, *p* = < 0.001). ODI was not significantly different. The operation time was shorter in MIDLIF (166.1 min vs. 196.2 min, *p* = 0.014). The facet joint violation is higher in MIS TLIF (21.6% vs. 2%, *p* = 0.009). The other complications were not significantly different including rate of implant removal, revision, and adjacent segment disease.

**Conclusion:**

In this study, postoperative VAS, operation time, and the rate of facet joint violation were significantly higher in the MIS TLIF group. Comparable outcomes were observed between MIDLIF and MIS TLIF in terms of fusion rate, time to fusion, and postoperative ODI score.

## Introduction

Transforaminal lumbar interbody fusion (TLIF) has been proven to be an effective solution for spinal instability [1, 2]. To minimize surgical trauma and enhance patient’s recovery, minimally invasive techniques are introduced in the past decades such as minimally invasive TLIF (MIS TLIF) [3]. MIS TLIF provides less estimated blood loss, less tissue trauma, and shorter hospital stays compared to traditional open TLIF [4, 5].

Pedicle screws used in MIS TLIF are placed in traditional trajectory. The traditional trajectory screw is inserted parallel to endplate, aims from lateral to medial, and placed convergently. Traditional trajectory screw provides well posterior fixation of spinal fusion. However, some disadvantages of traditional pedicle screw insertion including medial pedicle wall breaching [6], facet joint violation [7], damage of the medial branches of dorsal rami of spinal nerves (MBN) [8, 9], and higher screw loosening rate in osteoporotic patients [10, 11]. The stability of traditional trajectory screws is provided by dorsal cortex and the surrounding cancellous bone. Osteoporosis would attenuate the screw strength and lead to higher screw loosening rate [12–14].

In order to enhance the screw strength, screw insertion through cortical bone trajectory (CBT) was first introduced by Santoni et al. in 2009 [15]. CBT increased 30% pull-out strength and 70% insertion torque compared with traditional trajectory [15, 16]. With the new trajectory aims from medial to lateral and from caudal to cephalad, CBT can purchase more cortexes and provides more strength. The different trajectory also brings the advantages of less facet joint violation, less medial pedicle wall breaching, and less surgical trauma [17]. A meta-analysis revealed that CBT and traditional trajectory received similar fusion rate, whereas CBT was associated with less blood loss and shorter hospital stays than traditional trajectory [17]. Additionally, CBT screw insertion could be performed with laminectomy and decompression simultaneously through a posterior midline approach (MIDLIF). Due to the advantages of CBT, MIDLIF is gaining popularity recently. However, few studies directly compare MIDLIF to MIS TLIF. This study aimed to compare surgical outcomes, clinical outcomes, and complications between MIS TLIF and MIDLIF.

## Materials and methods

### Patients

The retrospective cohort study was approved by the Institutional Review Board of Show Chwan Memorial Hospital (No. 1100706). Eligible patients were those who underwent MIS TLIF between November 2014 and March 2018 (MIS TLIF group) or underwent MIDLIF (MIDLIF group) between April 2018 and April 2021 in the Show Chwan Memorial Hospital, had spinal instability due to degenerative or isthmic spondylolisthesis Meyerding grade I-II [18], fusion levels less than three, and received postoperative follow-up for at least one year. Patients were excluded if they had active infection, malignancy, prior history of spinal surgery, or postoperative follow-up for less than one year. All operations were performed by the one experienced spinal surgeon.

### Surgical techniques

#### MIS TLIF

Operation was performed using Wiltse approach as previously described [19]. A 4 cm incision was made on the cage insertion side. The dissection was made between multifidus and longissimus and down to the lamina and facet. Unilateral approach bilateral decompression was performed to relieve pressure on the spinal nerves in cases of spinal stenosis. The intervertebral body space was carefully prepared including disc material removal, decortication of bony endplate, autograft placement, and cage selection. Superior facet joint was preserved to prevent future adjacent segment disease. Staple wounds were made on the other side to facilitate percutaneous screwing. Pedicle screw was placed with guide pins and dilators under fluoroscopic guidance. Soft tissue around the entry point was not cauterized due to the limited operative field of the staple wound. After the procedure of decompression, cage placement and screw insertion were done, the rod were assembly and secured. Checked the final structure under fluoroscope before wound closure. The wound was closed layer by layer. The screws we used in MIS TLIF were MANTIS (Stryker, Kalamazoo, MI, USA) and Trend I systems (Biomech, Taipei, Taiwan). All polyetheretherketone cage (G cage, Biomech, Taipei, Taiwan) was used in MIS TLIF.

#### MIDLIF

MIDLIF was performed using CBT screw insertion as previously described [20, 21]. The midline incision was made and dissection was made between spinal process and paraspinal muscles. Exposed the lamina and facet. The bilateral entry point of the screw was exposed with electrocautery, which is different from MIS TLIF. The CBT screw was inserted divergently under fluoroscopy. The decompression was done on the symptomatic side and until the pulsation of the spinal cord restored. After the decompression and screw insertion were done, the rod and crosslink were assembled and secured. The final structure under fluoroscope was checked before wound closure. Wound was closed layer by layer. The CBT screws placed in MIDLIF were Wiltrom (Wiltrom, Hsinchu, Taiwan) and Trend II (Biomech, Taipei, Taiwan) systems. Interbody fusion was done using all polyetheretherketone cage (G cage, Biomech, Taipei, Taiwan).

### Data collections

Data were collected through retrospective chart review. Demographics, body mass index (BMI), bone marrow density (BMD), smoking status, comorbidity (including diabetes mellitus, hypertension, and coronary artery disease), diagnosis, perioperative data (i.e., operation time and blood loss), postoperative data (i.e., change in hemoglobin and hospital stay), surgical outcomes, clinical outcomes, and complications were collected.

Surgical outcomes included fusion status at postoperative one-year and time to bony fusion. Fusion status was assessed by computed tomography (CT) scan. The CT was arranged once the lumbar spine flexion extension radiograph showed the angular motion change less than 5 degree at fusion level, trabecular bony bridge formation without radiolucent line, and no implant failure [22, 23].

Clinical outcomes included pain degree and spine function, which were assessed using visual analog scale (VAS) and Oswestry Disability Index (ODI), respectively. The VAS score ranges from 0 to 10 (0 = least pain, 10 = worst pain). The ODI contains 10 patient-completed questions to evaluate spine function. Each question is scored on the scale of 0 to 5 (0 = best outcome, 5 = worst outcome). The overall ODI score ranges from 0 to 100% and a lower score indicates better function [24]. Evaluation of pain and spine function were performed at preoperative, postoperative 3-month, and postoperative 1-year.

**Fig. 1:**
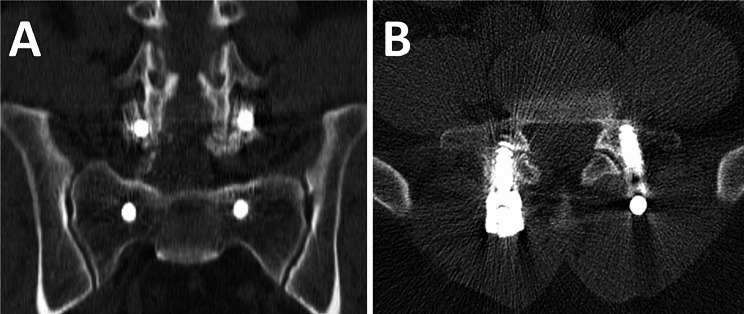
Coronal view (**A**) and axial view (**B**) of CT showing L5 right facet joint violation in a patient undergoing MIS TLIF

Complications included implant removal due to screw head irritation, revision surgery, screw loosening, and implants related complication. Implants related complications consisted of medial breaching, lateral breaching, and facet joint violation (Fig. 1). Screw loosening was defined as presence of radiolucent area of more than 1 mm surrounding the screw and double halo sign on lumbar spine radiograph [25]. The CT scan was arranged when symptomatic complications occurred. The screw malposition was investigated by authors using the fusion CT as mentioned above. Safe zone was defined as breaching less than 2 mm [26]. Screw breaching more than 2 mm were recorded. All the images were interpreted independently by two orthopedists. Disagreement of the interpretation was resolved by further discussion. Lumbar spine radiography was performed preoperatively, immediate postoperatively, and at postoperative 1-, 2-, 3-, 6, and 12-months.

### Statistical analysis

Continuous variables were presented as mean (standard deviation) and categorical variables as count (percentage). To compare the MIS TLIF and MIDLIF groups, Mann-Whitney U test and Fisher’s Exact test were used for continuous variables and categorical variables, respectively. One-year fusion rate was compared using log-rank test. A two-tailed *p* < 0.05 indicated statistical significance. All analyses were performed using IBM SPSS Statistics for Windows, version 24 (IBM Corporation, Armonk, NY, USA).

## Results

A total of 87 patients were included in this study, 37 in the MIS TLIF group and 50 in the MIDLIF group. There were no significant differences between the two groups regarding age, gender, BMI, smoking, chronic diseases, pathology, and preoperative spondylolisthesis grade. There were lower BMD (0.885 vs. 0.697, *p* = 0.002) and more 2-level fusion (24.3 vs. 46%, *p* = 0.038) in MIDLIF (Table 1).

**Table 1:** Demographics and baseline characteristics between the patients undergoing MIS TLIF and those who underwent MIDLIF

	MIS TLIF	MIDLIF	*P*
Number of patients	37	50	
Gender			0.477
Male	13 (35.1)	14 (28)	
Female	24 (64.9)	36 (72)	
Age (year)	60.0 (15.2)	65.0 (13.8)	0.115
BMI	26.8 (5.5)	26.4 (4.9)	0.703
BMD	0.885 (0.17)	0.679 (0.172)	0.002
Smoking	7 (18.9)	5 (10)	0.233
Comorbidity (including DM, HTN, and CAD)	16 (43.2)	25 (50)	0.533
Pathology			0.133
Degenerative spondylolithesis	29 (78.4)	45 (90)	
Isthmic spondylolithesis	8 (21.6)	5 (10)	
Preoperative Meyerding grade			0.857
I	20 (54.1)	28 (56)	
II	17 (45.9)	22 (44)	
Number of fusion levels			0.038*
1	28 (75.7)	27 (54)	
2	9 (24.3)	23 (46)	

Data were presented as count (percentage) or mean (standard deviation)

Abbreviation: MIS TLIF, minimally invasive transforaminal lumbar interbody fusion; MIDLIF, midline lumbar interbody fusion; BMI, body mass index; BMD. bone mineral density; DM, diabetes mellitus; HTN, hypertension; CAD, coronary artery disease

As presented in Table 2, the operation time (196.2 vs. 166.1, *p* = 0.014) was shorted in MIDLIF. No significant differences in blood loss, change in hemoglobin, and hospital stay were observed. These two groups had similar time to fusion and one-year fusion rate (Table 2).

**Table 2:** Operative data and surgical outcomes between the patients undergoing MIS TLIF and those who underwent MIDLIF

	MIS TLIF	MIDLIF	*P*
Number of patients	37	50	
Operation time (minute)	196.2 (69.1)	166.1 (42.5)	0.014
Blood loss (ml)	332.0 (323.4)	309.8 (237.9)	0.713
Change in hemoglobin (mg/dL)	-2.7 (0.9)	-2.7 (0.9)	0.838
Hospital stays (day)	7.2 (2.1)	6.5 (2.5)	0.203
Time to fusion (day)	166.1 (62.6)	188.4 (80.5)	0.817
Fusion at postoperative one-year	36 (97.3)	46 (92)	0.559

Data were presented as count (percentage) or mean (standard deviation)  Abbreviation: MIS TLIF, minimally invasive transforaminal lumbar interbody fusion; MIDLIF, midline lumbar interbody fusion

**Fig. 2:**
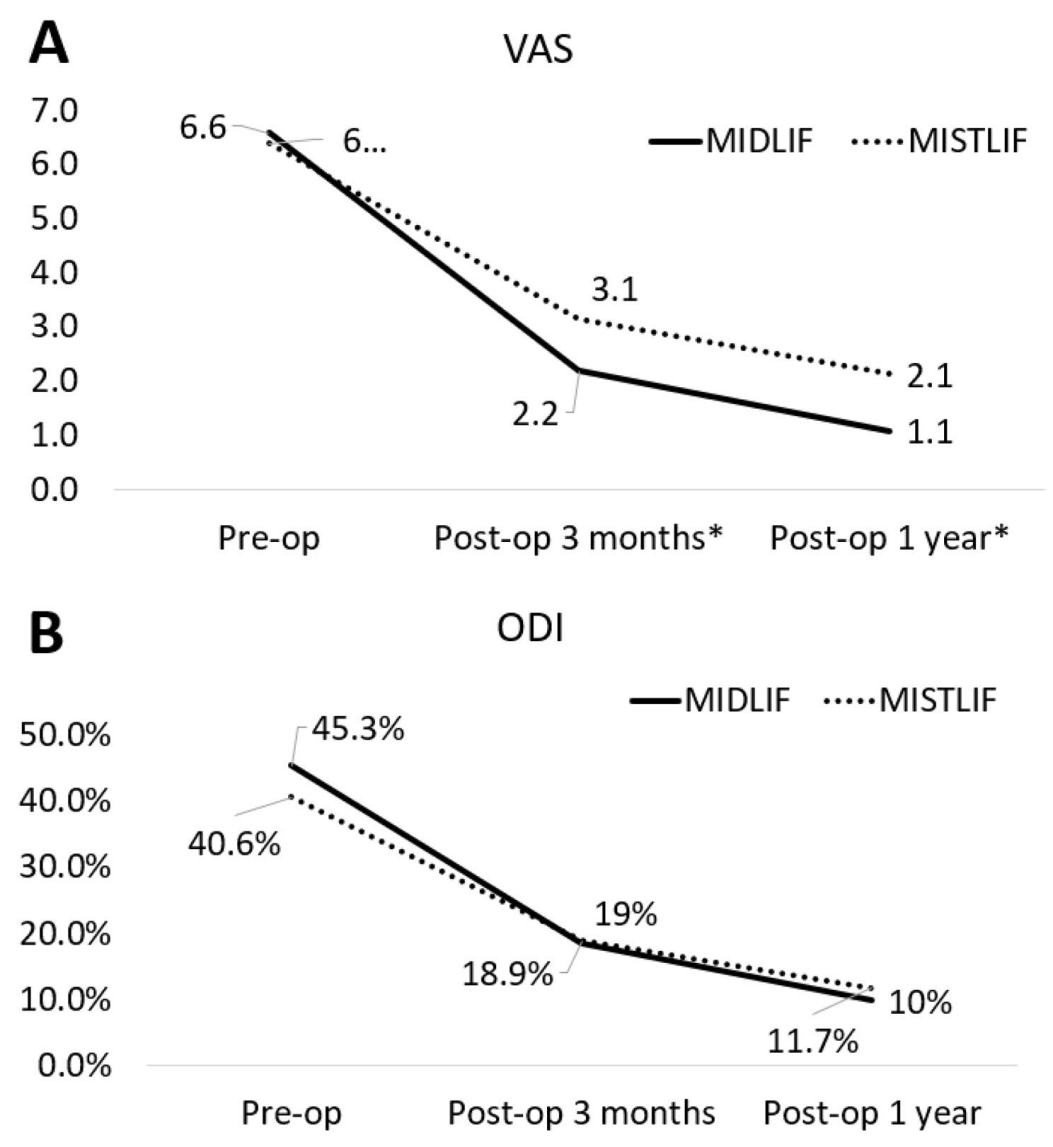
Changes over time in VAS (**A**) and ODI (**B**) scores between patients undergoing MIS TLIF and those who underwent MIDLIF. *Asterisks indicated statistical significance between the two groups (*P* < 0.05)

Both groups had improved VAS and ODI postoperatively. The MIDLIF group had significantly lower VAS at postoperative 3-months (2.2 vs. 3.1, *p* = 0.002) and postoperative 1-year (1.1 vs. 2.1, *p* < 0.001). ODI was not significantly different between the two groups during postoperative follow-up (Fig. 2).

### Complications

No significant difference was observed between the two groups regarding implant removal, revision, and adjacent segment disease. The facet joint violation (21.6% vs. 2%, *p* = 0.009) was higher in MIS TLIF (Table 3). Medial breaching was also higher in MIS TLIF, but the difference was not statistically significant.

**Table 3:** Complications between the patients undergoing MIS TLIF and those who underwent MIDLIF

	MIS TLIF	MIDLIF	*P*
Number of patients	37	50	
Implant removal	2 (5.4)	0 (0)	0.347
Revision	2 (5.4)	1 (2)	0.790
Adjacent segment disease	2 (5.4)	1 (2)	0.790
Screw loosening rate	3 (8.1)	6 (12)	0.816
Implant related complication			
Medial breaching	6 (16.2)	3 (6)	0.234
Lateral breaching	0	1 (2)	0.879
Facet joint violation	8 (21.6)	1 (2)	0.009

Data were presented as count (percentage)

Abbreviation: MIS TLIF, minimally invasive transforaminal lumbar interbody fusion; MIDLIF, midline lumbar interbody fusion

*Screw malposition, including breaching and facet joint violation, was determined by CT

Five patients experienced reoperation, four in the MIS TLIF group and one in the MIDLIF group. The duration until reoperation ranged from 2 days to 34.4 months. The indications of reoperation included symptomatic medial screw breaching, facet joint violation with nonunion, and screw head irritation. Two patients received revision at postoperative 2-day due to symptomatic screw breaching, one in each group. Three patients presented with chronic low back pain postoperatively in MIS TLIF. Two were diagnosed screw head irritation and one facet joint violation with nonunion. The symptoms were resolved after the operations (Table 4).

**Table 4:** Reoperations in the patients undergoing MIS TLIF and those who underwent MIDLIF

No.	Group	Initial fusion level	Indication	Treatment	Duration to reoperation
1	MIS TLIF	L4-S1	Symptomatic medial breaching	Revision	2 days
2	MIS TLIF	L4-5	Screw head irritation	Implant removal	20.1 months
3	MIS TLIF	L4-S1	Screw head irritation	Implant removal	34.4 months
4	MIS TLIF	L5-S1	Facet joint violation and nonunion	Revision	25.9 months
5	MIDLIF	L4-5	Symptomatic medial breaching	Revision	2 days

Abbreviation: MIS TLIF, minimally invasive transforaminal lumbar interbody fusion; MIDLIF, midline lumbar interbody fusion

## Discussion

Although both MIS TLIF and MIDLIF are common surgical approaches for spinal disorders, evidence of directly comparing MIS TLIF and MIDLIF is limited. In this study, there were no significant differences in one-year fusion rate, time to fusion, and improvement of spinal function between MIS TLIF and MIDLIF, except that MIDLIF provided better effect of pain relief than MIS TLIF at postoperative 3-month and one-year. Other complications were comparable. The MIS TLIF group had numerical higher incidence of implant removal, and revision than the MIDLIF group.

**Fig. 3:**
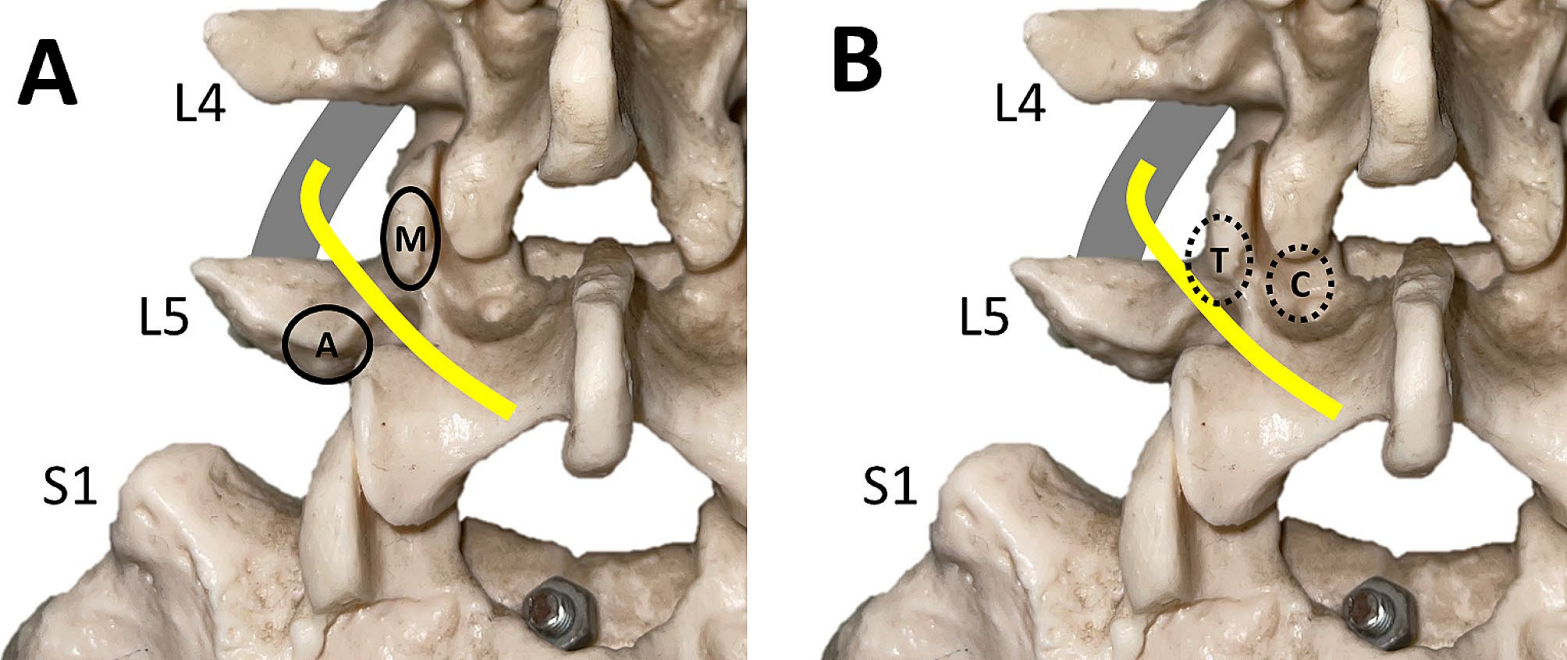
Anatomy of medial branch of dorsal ramus nerve (**A**) and anatomical relationship among entry point of cortical bone trajectory, entry point of traditional trajectory, and medial branch of dorsal ramus nerve (**B**). Gray line: L4 nerve root; yellow line: medial branch of dorsal ramus of spinal nerve; M: mamillary process; A: accessory process; C: entry point of cortical bone trajectory; T: entry point of traditional trajectory

The higher postoperative pain score and incidence of implant removal in the MIS TLIF group may be related with MBN-induced back pain after spinal instrumentation [27, 28]. MBN lies between facet and transverse process [29, 30] and is fixed by the strong fibers of mammillo-accessory ligament, which extends between the mammillary process and accessory process (Fig. 3A) [9]. A cadaveric study by Regev et al. compared MBN injury after mini-open versus percutaneous pedicle screw insertion [8]. MBN transection was observed in 84% of the pedicles when using mini-open technique and in 20% of the pedicles when the screw was placed via percutaneous approach (*P* < 0.01). When the MBN is transected or ablated during pedicle screw insertion, there would be less MBN-related postoperative pain. Conversely, when performing percutaneous screw insertion via traditional trajectory, the screw head is just beside the intact MBN. Thus, it might result in nerve impingement or irritation, contributing to postoperative back pain (Fig. 3B). In MIDLIF, soft tissues around the entry point are ablated, which may damage the MBN. In MIS TLIF, the MBN is relatively preserved due to percutaneous insertion technique. The difference in entry points and soft tissue preservation around entry points may lead to greater postoperative pain with MIS TLIF.

Our results revealed that both MIDLIF and MIS TLIF groups had one-year fusion rate of over 90%, which were comparable with previous reports [31–33]. Several studies also observed higher fusion rate in MIDLIF than in MIS TLIF [31–33]. The greater proportion of two-level fusion in the MIDLIF versus MIS TLIF group (40.7% vs. 24.3%) lead to lower one-year fusion rate and longer time to fusion but none of these were statistically significant. Most of previous studies focused on the patient undergoing one-level spinal fusion [32–35] or included only a few patient with two-level fusion [31]. By contrast, this study included more patients with two-level fusion, which indicated more complicated nature of the patients.

This study revealed similar blood loss between two groups. The operation time was statistically faster in the MIDLIF group even with a larger proportion of two-level fusion. Previous reports [32–34] also showed shorter operation time in MIDLIF. The narrow surgical field of view and high technical demands of MIS TLIF increase operation time, especially when resecting contralateral lesions. On the other hand, MIDLIF is performed via a posterior midline incision and bilateral lesions could be approached more easily.

Our study observed a trend towards a lower complication rate for MIDLIF compared to MIS TLIF, although the difference was not statistically significant. The study by Wu et al. revealed similar results that overall complication rate was lower in MIDLIF than in MIS TILF (18.75% vs. 29.4%, *P* = 0.423). Kasukawa et al. reported higher rate of correct screw positioning in MIDLIF than in MIS TILF (90% vs. 84.1%). The higher malposition rate in MIS TILF might be related with the instinct characteristic of traditional trajectory, in which screws are placed convergently and entry points are just nearby the facet joints.

A meta-analysis published by Hu et al. indicated that no difference was found in VAS score when comparing MIDLIF and other posterior fusion technique [17]. A study published by Wu et al. better VAS leg pain at post operative 6 months but no difference found at 1 year follow up [33]. In our study, we revealed the same tendency and the better VAS score at post operative 3 months and 1 year were noted.

This study had limitations. The first one came from the retrospective study design. Potential selection bias and reporting bias could not be avoided. All patients were operated in the same hospital. The single-institutional results may not be applicable in other institutions, which limited the external validity of this study. Additionally, all patients were postoperatively followed for at least one year. However, some long-term complications, such as adjacent segment disease, might not be thoroughly observed. Furthermore, there were more two-level fusion done in MIDLIF, which indicated the severity of the patient was not evenly distributed and may produce bias.

In conclusion, this study observed comparable one-year fusion rate, time to fusion, function improvement, and complications between the patients receiving MIS TLIF and MIDLIF. MIDLIF provided better pain relief at postoperative 3-months and one-year. Further large-scale studies are warranted for identifying the patients who would benefit most from MIS TLIF and MIDLIF respectively.

## Data Availability

All data generated or analysed during this study are included in this published article.

## Abbreviations

BMD: Bone marrow density
BMI: Body mass index
CBT: Cortical bone trajectory
CT: Computed tomography
MIDLIF: Midline lumbar interbody fusion
MIS TLIF: Minimally invasive transforaminal lumbar interbody fusion
ODI: Oswestry Disability Index
TLIF: Transforaminal lumbar interbody fusion
VAS: Visual analog scale
